# Supplementary material for: A way to break bones? The weight of intuitiveness
Source: PLoS One. 2021 Oct 29;16(10):e0259136. doi: 10.1371/journal.pone.0259136 (PMC8555848; doi:10.1371/journal.pone.0259136)
Supplement: S1 Appendix — (DOCX) [file pone.0259136.s001.docx]

Apendix I


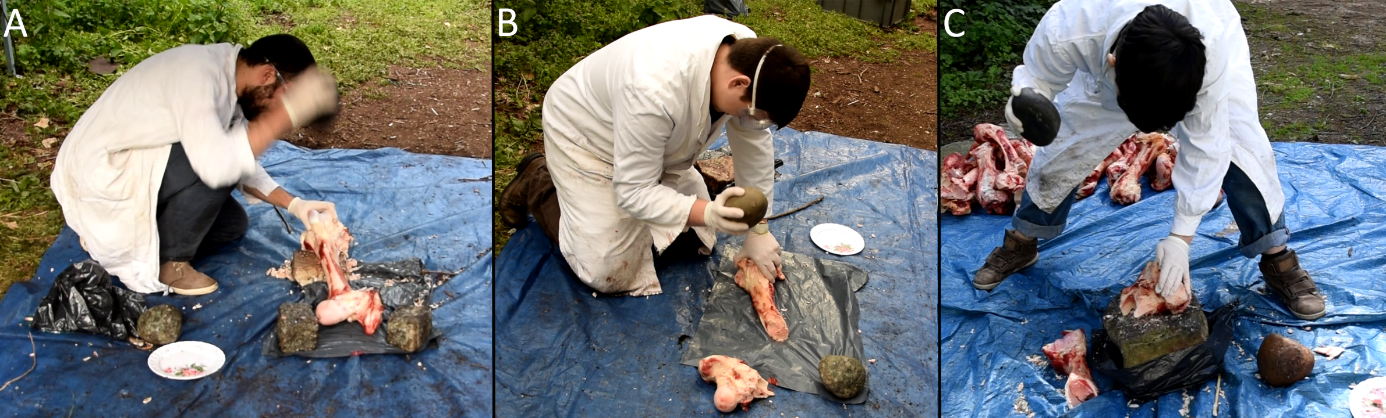


S1 Figure: Example of individual position during the breakage. A-individual n°10 squatting, B-individual 8 kneeling and C-individual n°1 standing.


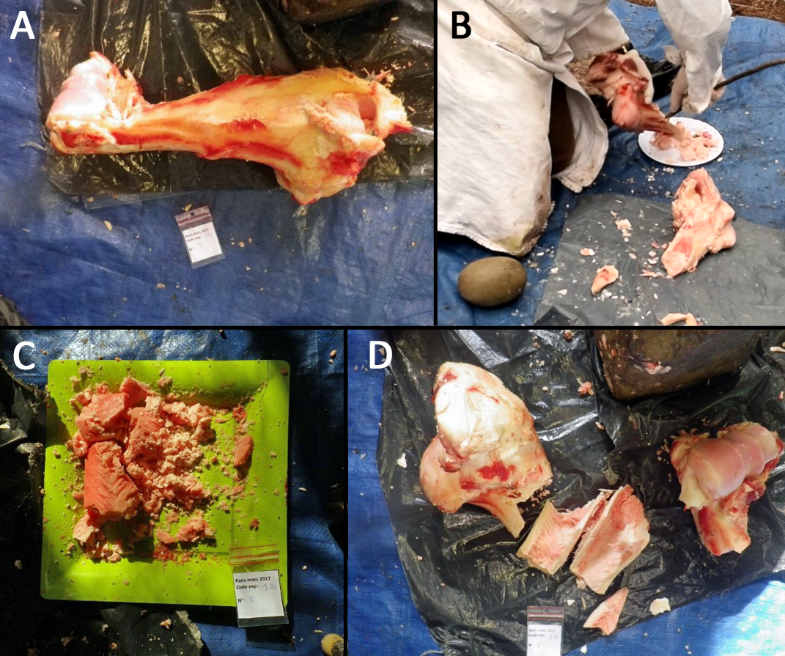


S2 Figure: Bone number 5 of individual n°7 (A, C and D) and Individual 8 (B); A-before breakage, B-yellow marrow recovery with wood stick, C-marrow recovered and D-bone remains after breakage.


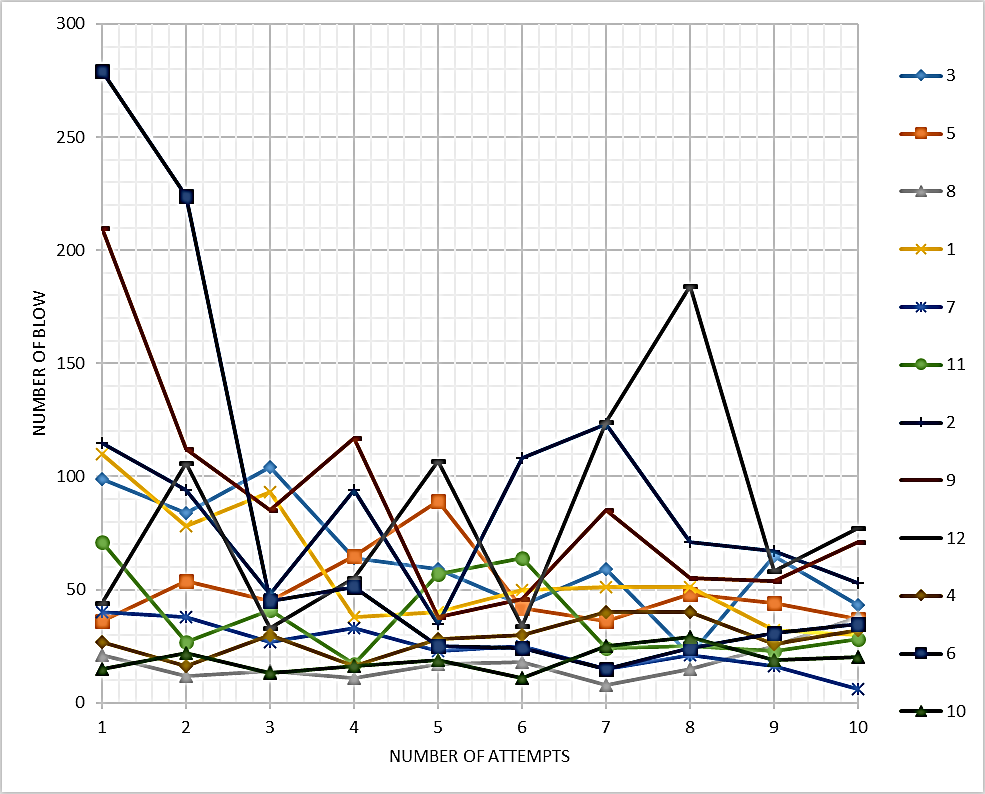


S3 Figure: The number of blows according to the number of try for each individual.


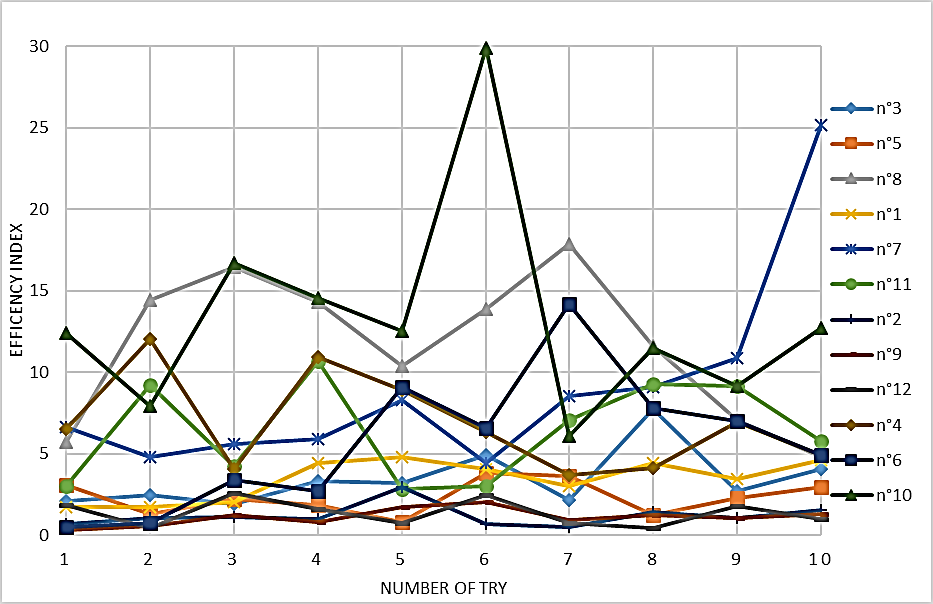


S4 Figure: Efficiency Index by individual during the experiment for each bone.


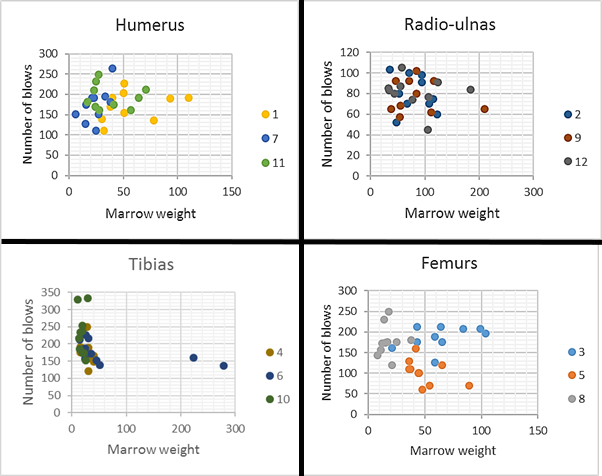


S5 Figure: Graph of the number of blows by the marrow weight (in gram) for each element


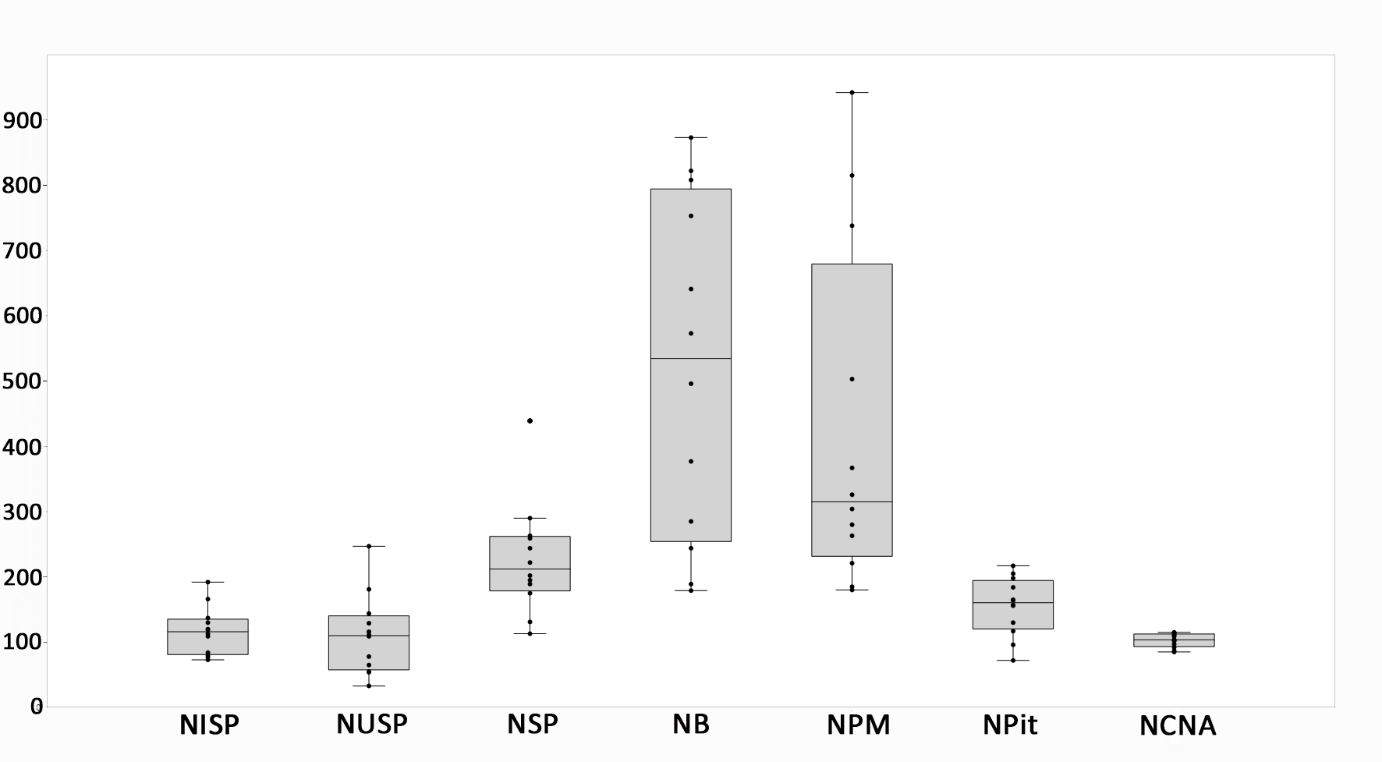


S6 Figure: Box plots and jitter with outsider of marrow Weight (MW), Number of Specimen (NSP), Number of identified specimens (NISP), Number of undetermined specimens (NUSP), Number of blows (NB), Number of percussion marks (NPM), Number of pit and grooves (Npit) and Number of crushing marks, of adhering flakes and of notches (NCNA).


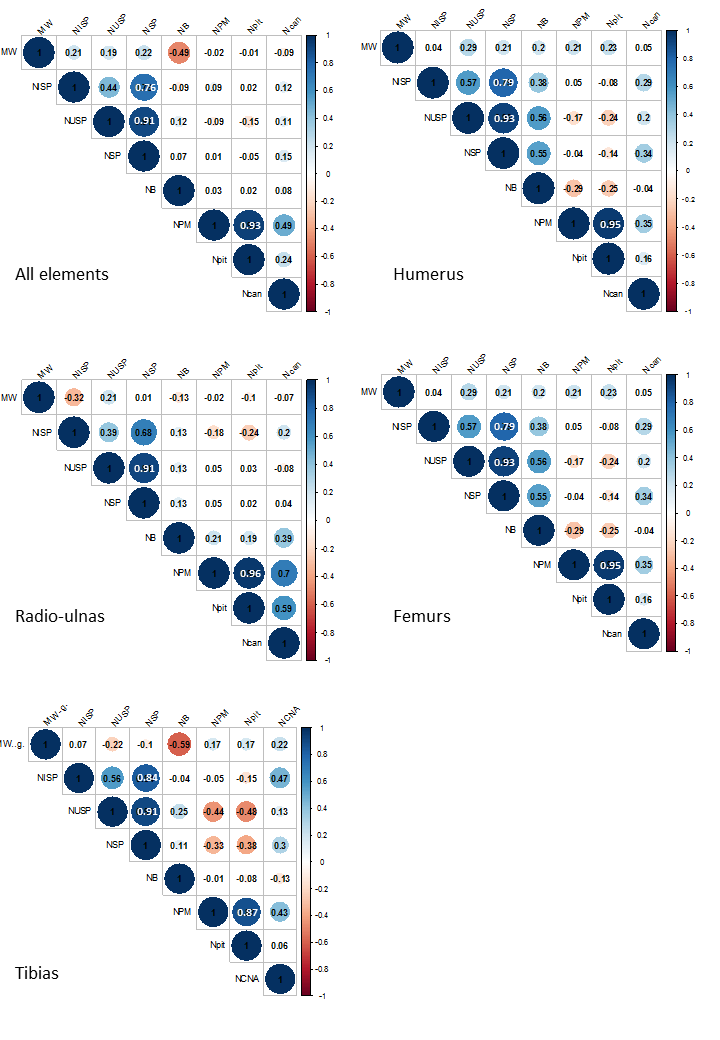


S7 Figure: Spearman's rank correlation coefficient results between the extracted marrow Weight (MW), Number of Specimen (NSP), Number of identified specimens (NISP), Number of undetermined specimens (NUSP), Number of blows (NB), Number of percussion marks (NPM), Number of pit and grooves (Npit) and Number of crushing marks, of adhering flakes and of notches (NCNA) for all elements, and each elements separately.


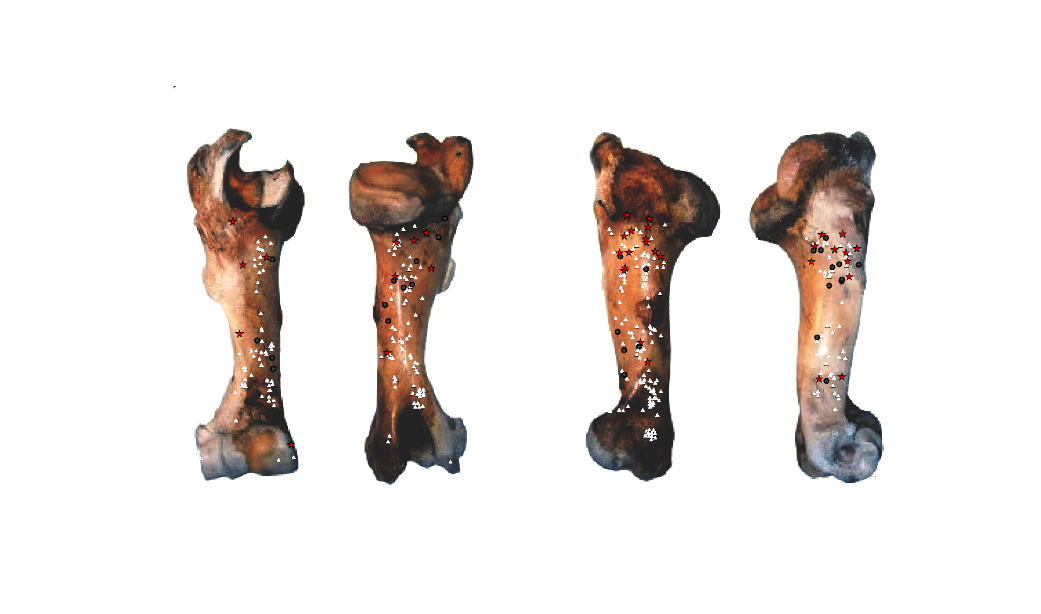

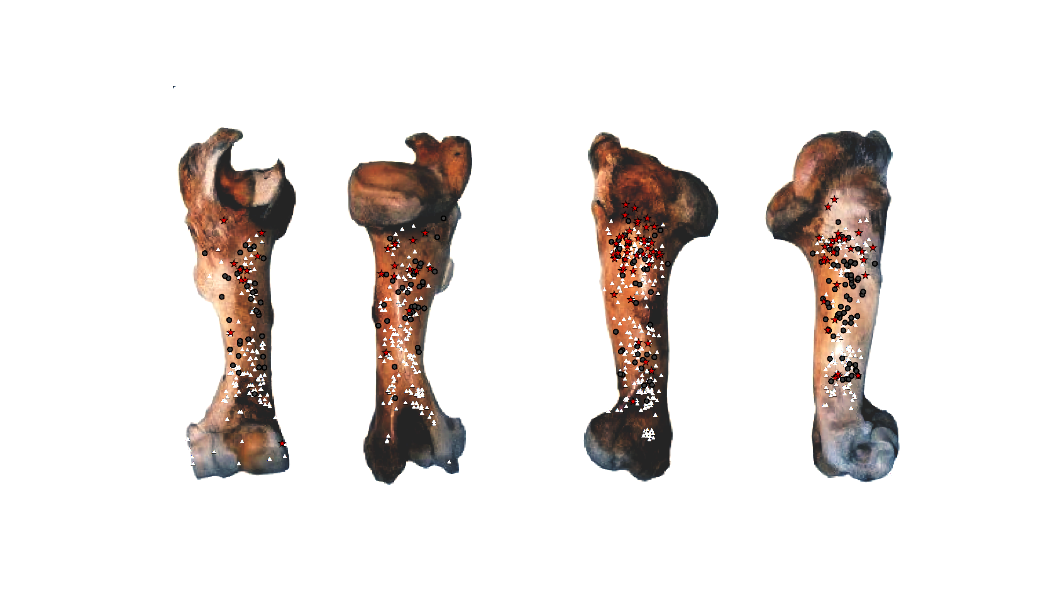


C

Ant. Post. Med. Lat.

Ant. Post. Med. Lat.

D


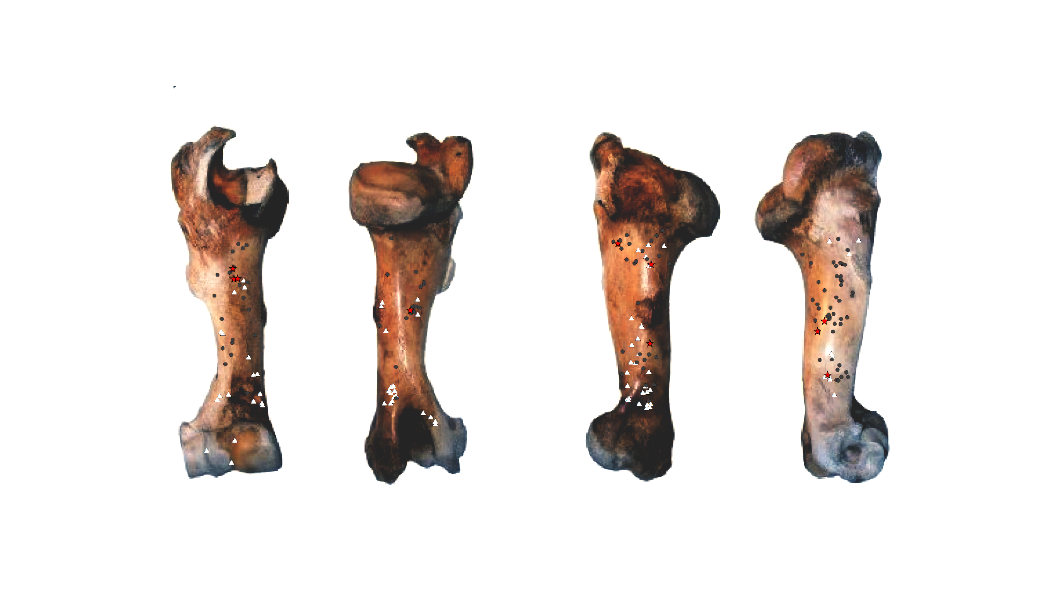

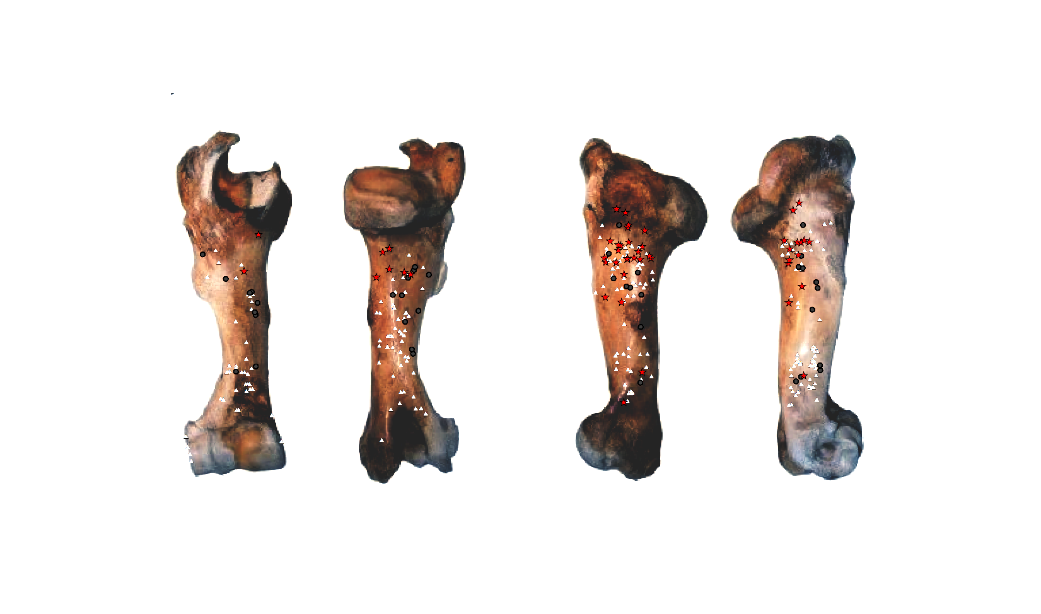


Ant. Post. Med. Lat.

Ant. Post. Med. Lat.

A

B

S7 Figure: Distribution of percussion marks along the humerus divided by type of percussion mark (red star: crushing marks, white triangle: pits and grooves and grey circle: notches and adhering flakes); A- Individual n°1; B- Individual n°7; C- Individual n°11; D- Merged humerus series.

S8 Figure: Distribution of percussion marks along the radio-ulnas divided by type of percussion mark (red star: crushing marks, white triangle: pits and grooves and grey circle: notches and adhering flakes); A- Individual n°2; B- Individual n°9; C- Individual n°12; D- Merged radio-ulnas series.


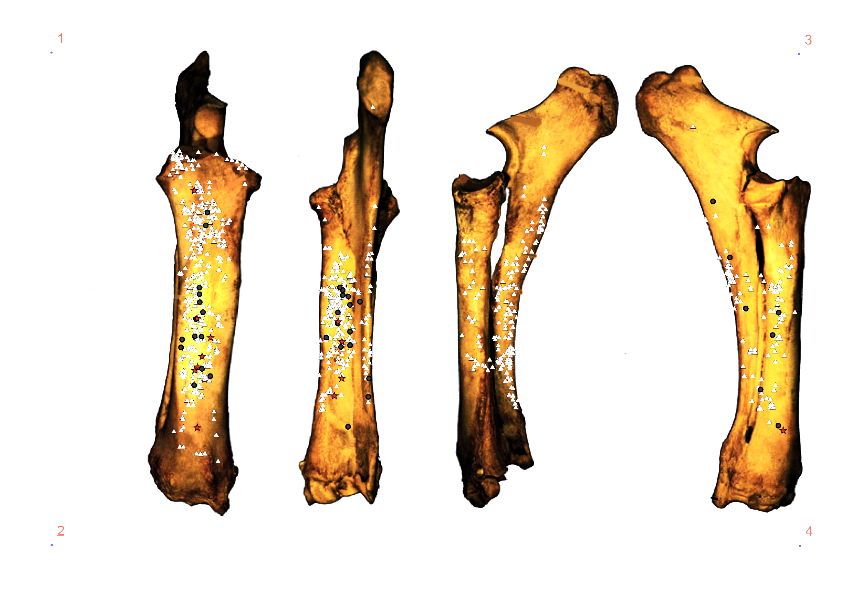

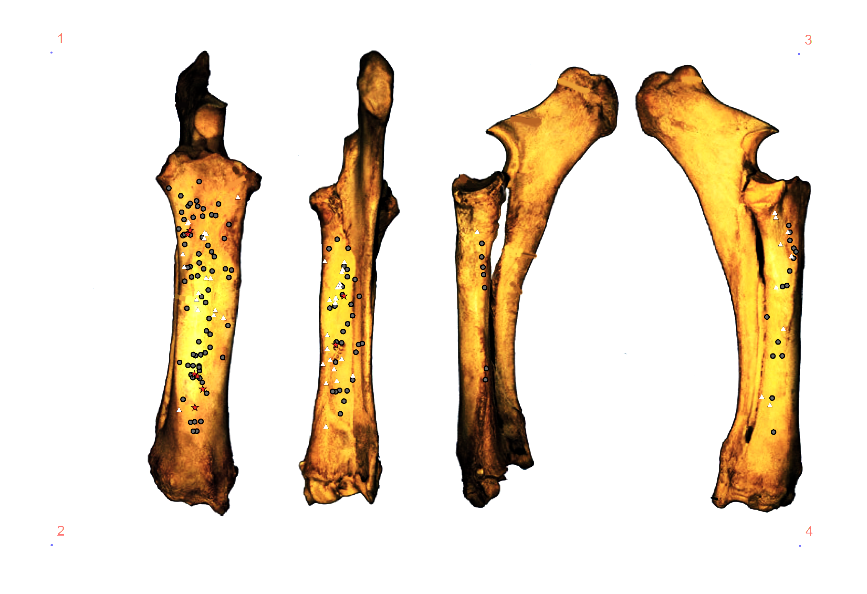

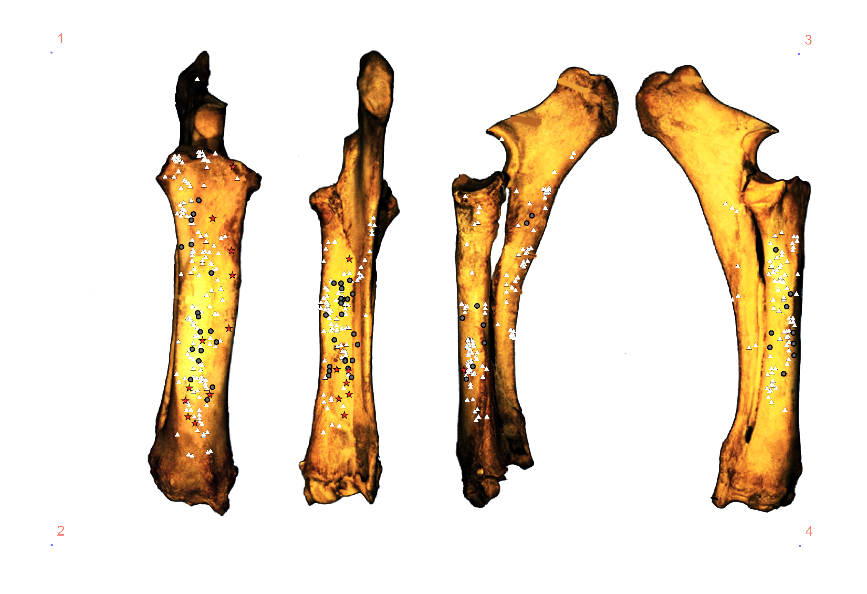

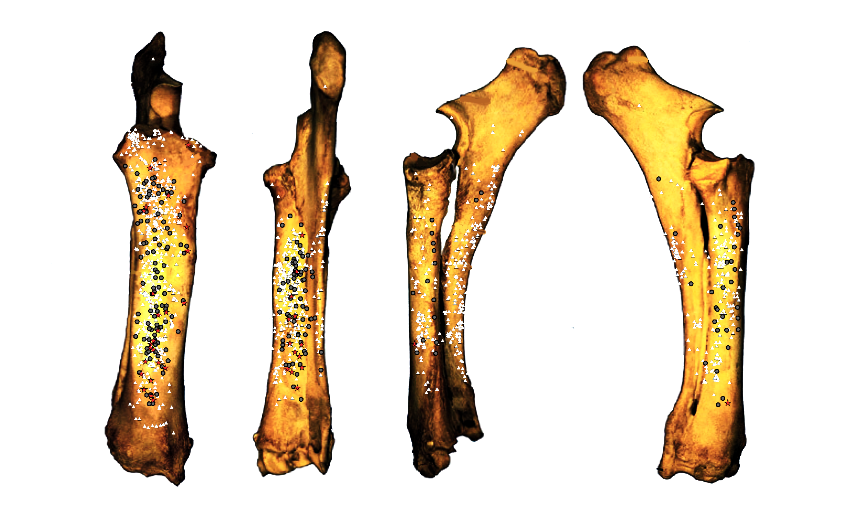


A

B

C

D

Ant. Post. Med. Lat.

Ant. Post Med. Lat.

Ant. Post. Med. Lat.

Ant. Post. Med. Lat.


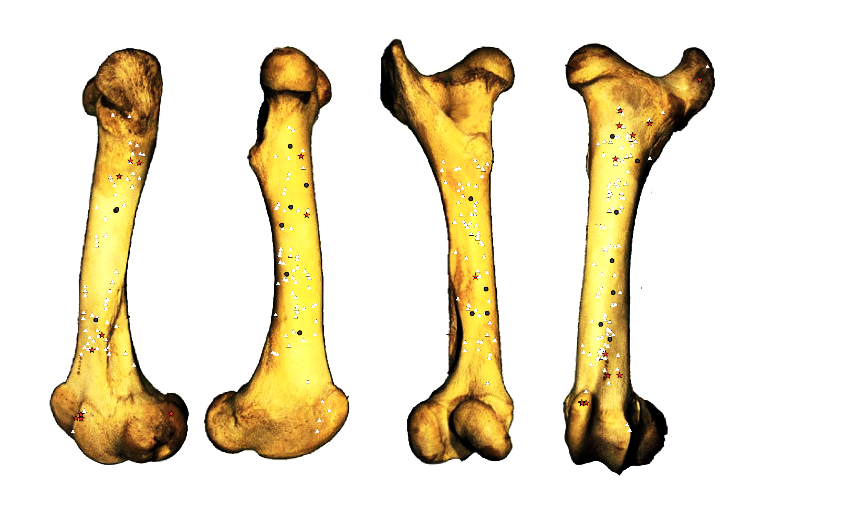

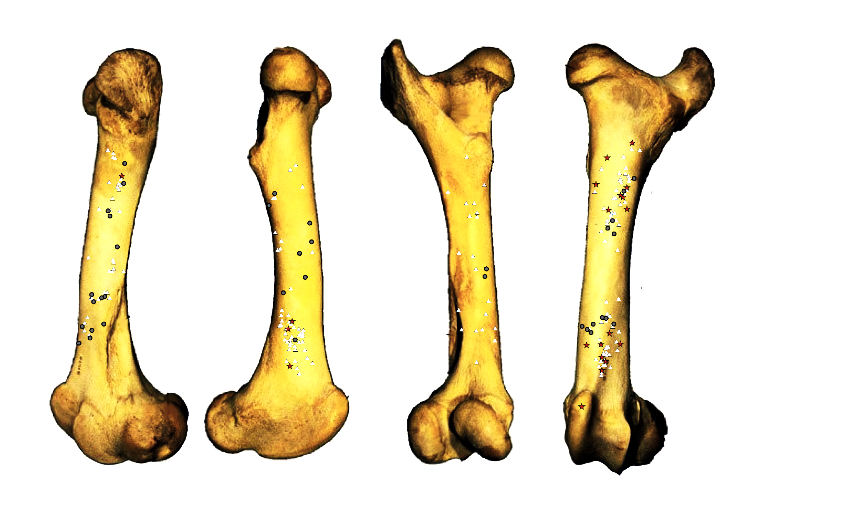

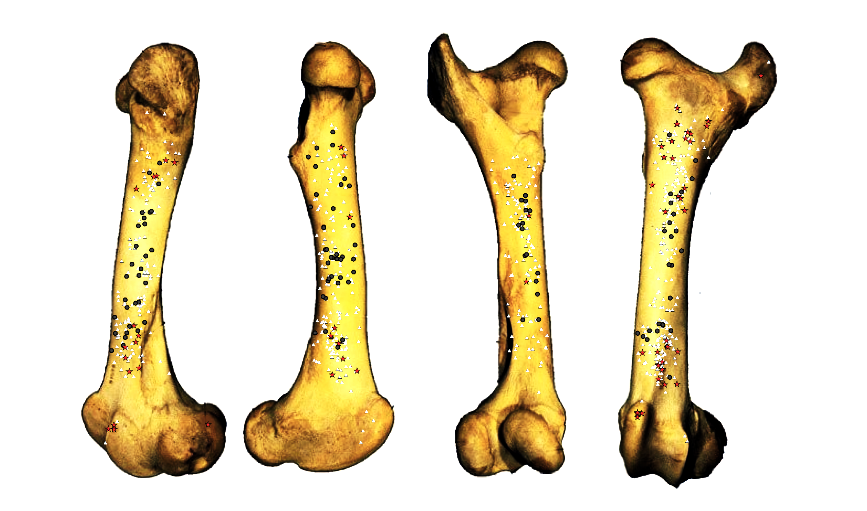

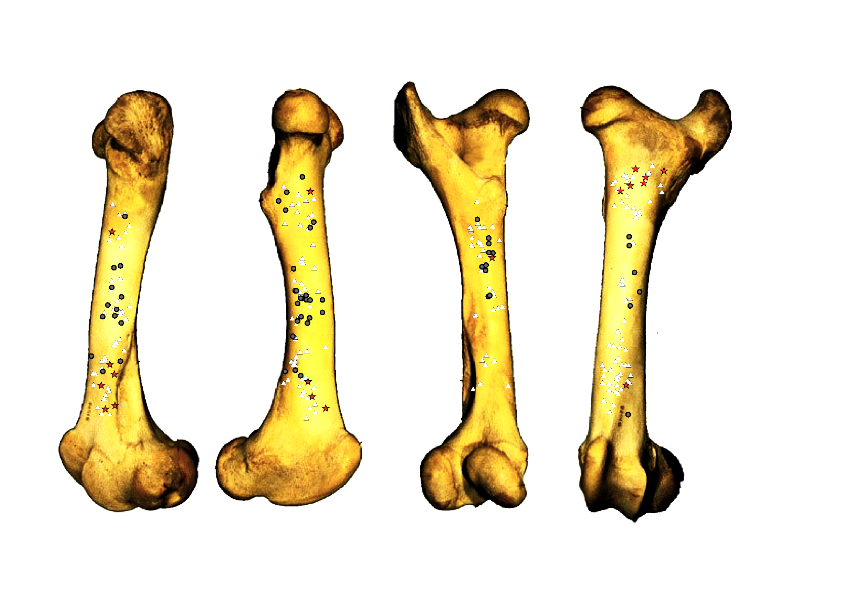


A

B

C

D

Ant. Post. Med. Lat.

Ant. Post. Med. Lat.

Ant. Post. Med. Lat.

Ant. Post. Med. Lat.

S10 Figure: Distribution of percussion marks along the femurs divided by type of percussion mark (red star: crushing marks, white triangle: pits and grooves and grey circle: notches and adhering flakes); A- Individual n°3; B- Individual n°5; C- Individual n°7; D-: Merged femurs.


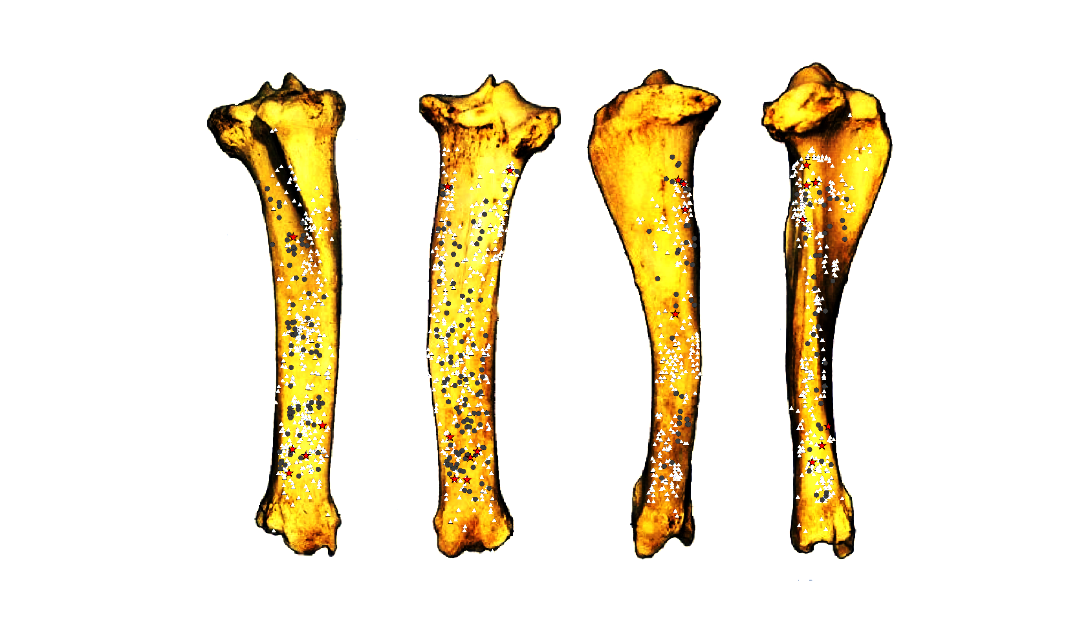

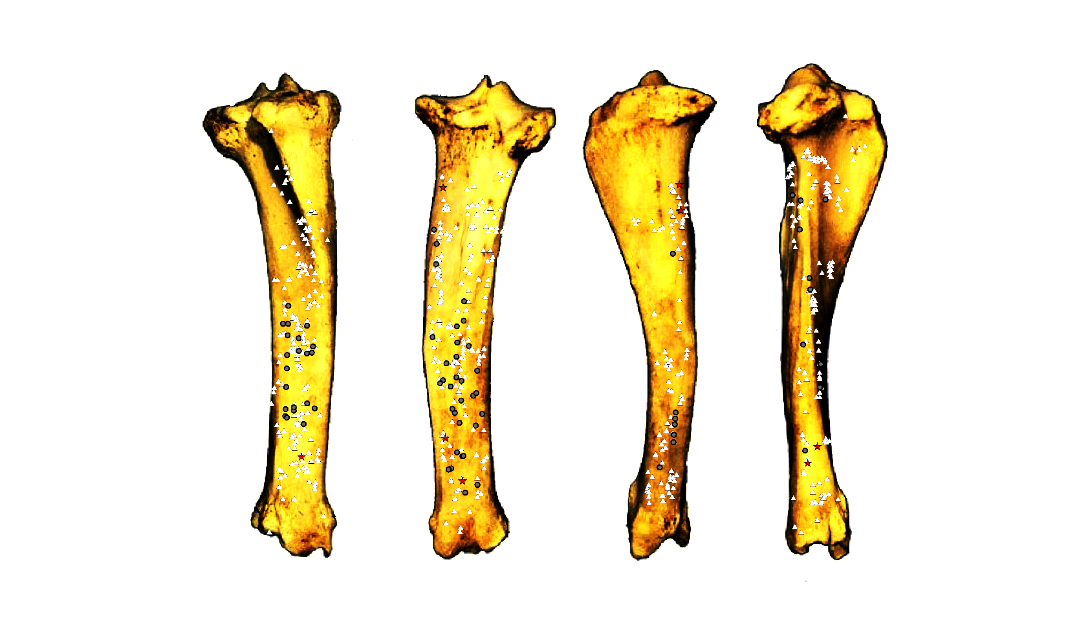

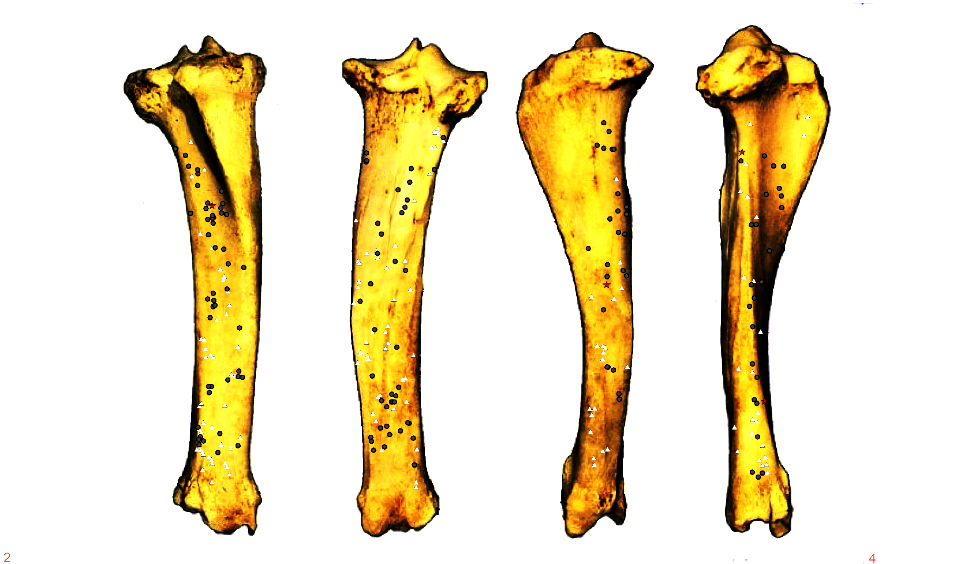

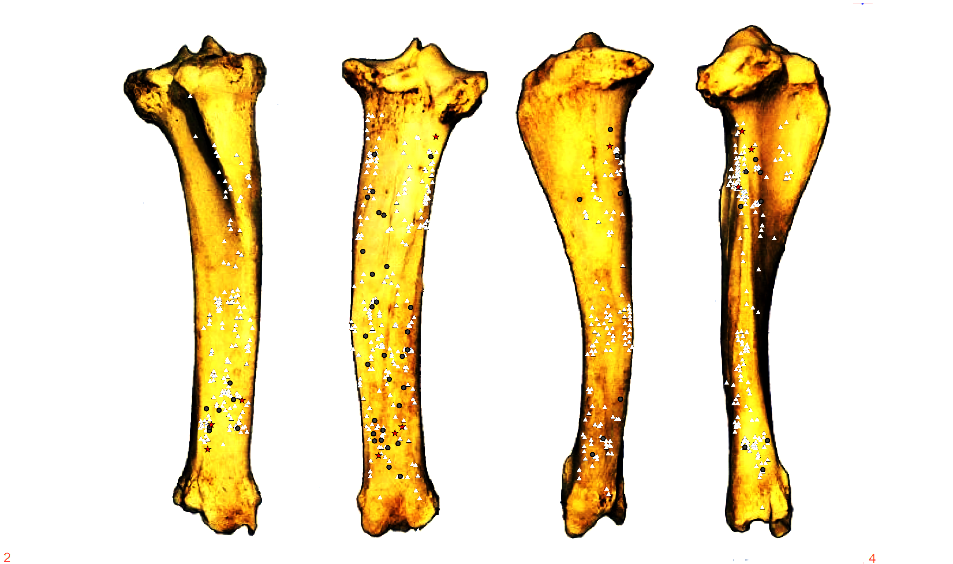


A

B

C

D

Ant. Post. Med. Lat.

Ant. Post. Med. Lat.

Ant. Post. Med. Lat.

Ant. Post. Med. Lat.

S11 Figure: Distribution of percussion marks along tibias divided by type of percussion mark (red star: crushing marks, white triangle: pits and grooves and grey circle: notches and adhering flakes A- Individual n°4; B- Individual n°6; C- Individual n°10; D- Merged tibias series.

S1 Table: The number of blows and the Efficiency Index, for each individual, regarding 50/50 the five first tries (T1) and the five last ones (T2), 3/-/3 the three first tries (T1) and the three last ones (T2).

|  |  | All Element | Humerus | Radio-ulna | Tibia | Femur |
| --- | --- | --- | --- | --- | --- | --- |
| Number of blows (5/5) | Degree of freedom | 11 | 2 | 2 | 2 | 2 |
|  | Wilcoxon signed rank test | 59 | 6 | 3 | 3 | 5 |
|  | P-value | 0.1294 | 0.25 | 1 | 1 | 0.5 |
| Number of blows  (3/-/3) | Degree of freedom | 11 | 2 | 2 | 2 | 2 |
|  | Wilcoxon signed rank test | 61 | 6 | 4 | 3 | 4 |
|  | P-value | 0.09229 | 0.25 | 0.75 | 1 | 0.75 |
| Efficiency Index (5/5) | Degree of freedom | 11 | 2 | 2 | 2 | 2 |
|  | Wilcoxon signed rank test | 21 | 0 | 3 | 2 | 2 |
|  | P-value | 0.1763 | 0.25 | 1 | 0.75 | 0.75 |
| Efficiency Index  (3/-/3) | Degree of freedom | 11 | 2 | 2 | 2 | 2 |
|  | Wilcoxon signed rank test | 26 | 0 | 3 | 3 | 4 |
|  | P-value | 0.3394 | 0.25 | 1 | 1 | 0.75 |

S2 Table: Results of Wilcoxon signed rank test of the Number of blows and the Efficiency Index between the first five attempts and the last five ones (5/5) and between the first three attempts and the last three ones (3/-/3).

S1 Dataset: Dataset of observations during the intuitive experiment of long bone breakage. figshare. Dataset. <https://doi.org/10.6084/m9.figshare.12709592>

S2 Dataset: Zooarchaeological dataset of observations during the analyses of long bone remains coming from the intuitive experiment of long bone breakage. figshare. Dataset. <https://doi.org/10.6084/m9.figshare.12709709.v2>

S3 Dataset: GIS coordinates of percussion marks for each bone series recorded during zooarchaeological analyses on the remains of long bones after the experiment and cleaning. Skeletal element, individual number, bone number, remain number, percussion marks number, coordinates XY. Percussion mark type are 1: Notch ; 2: notch with adhering flake ; 3: ovoid pit ; 4: triangular pit ; 6: crushing mark. figshare. Dataset. <https://doi.org/10.6084/m9.figshare.12896852.v1>

Supporting Information 16: Experiment protocol: Delphine Vettese 2020. An archaeological experiment focused on the intuitive way of long bones breakage to extract marrow. **protocols.io** [dx.doi.org/10.17504/protocols.io.be7fjhjn](https://www.protocols.io/view/an-archaeological-experiment-focused-on-the-intuit-be7fjhjn/dx.doi.org/10.17504/protocols.io.be7fjhjn)
