# Supplementary material for: A way to break bones? The weight of intuitiveness
Source: PLoS One. 2021 Oct 29;16(10):e0259136. doi: 10.1371/journal.pone.0259136 (PMC8555848; doi:10.1371/journal.pone.0259136)
Supplement: Attachment — Submitted filename: Response to Reviewers.docx [file pone.0259136.s002.docx]

Haut du formulaire

Bas du formulaire

| Date: | Jul 27 2021 12:04PM |
| --- | --- |
| To: | "Delphine Vettese" delphinevettese@aol.com |
| From: | "PLOS ONE" plosone@plos.org |
| Subject: | PLOS ONE Decision: Revision required [PONE-D-21-06808R1] |

PONE-D-21-06808R1
A way to break bones? The weight of intuitiveness
PLOS ONE

Dear Dr. Vettese,

Thank you for submitting your manuscript to PLOS ONE. After careful consideration, we feel that it has merit but does not fully meet PLOS ONE’s publication criteria as it currently stands. Therefore, we invite you to submit a revised version of the manuscript that addresses the points raised during the review process.

Please submit your revised manuscript by September 30, 2021. If you will need more time than this to complete your revisions, please reply to this message or contact the journal office at plosone@plos.org. When you're ready to submit your revision, log on to https://www.editorialmanager.com/pone/ and select the 'Submissions Needing Revision' folder to locate your manuscript file.

Please include the following items when submitting your revised manuscript:

- A rebuttal letter that responds to each point raised by the academic editor and reviewer(s). You should upload this letter as a separate file labeled 'Response to Reviewers'.
- A marked-up copy of your manuscript that highlights changes made to the original version. You should upload this as a separate file labeled 'Revised Manuscript with Track Changes'.
- An unmarked version of your revised paper without tracked changes. You should upload this as a separate file labeled 'Manuscript'.

If you would like to make changes to your financial disclosure, please include your updated statement in your cover letter. Guidelines for resubmitting your figure files are available below the reviewer comments at the end of this letter.

If applicable, we recommend that you deposit your laboratory protocols in protocols.io to enhance the reproducibility of your results. Protocols.io assigns your protocol its own identifier (DOI) so that it can be cited independently in the future. For instructions see: <http://journals.plos.org/plosone/s/submission-guidelines#loc-laboratory-protocols>. Additionally, PLOS ONE offers an option for publishing peer-reviewed Lab Protocol articles, which describe protocols hosted on protocols.io. Read more information on sharing protocols at <https://plos.org/protocols?utm_medium=editorial-email&utm_source=authorletters&utm_campaign=protocols>.

We look forward to receiving your revised manuscript.

Kind regards,

Enza Elena Spinapolice, Ph.D
Academic Editor
PLOS ONE

Journal Requirements:

Please review your reference list to ensure that it is complete and correct. If you have cited papers that have been retracted, please include the rationale for doing so in the manuscript text, or remove these references and replace them with relevant current references. Any changes to the reference list should be mentioned in the rebuttal letter that accompanies your revised manuscript. If you need to cite a retracted article, indicate the article’s retracted status in the References list and also include a citation and full reference for the retraction notice.

Additional Editor Comments (if provided):


[Note: HTML markup is below. Please do not edit.]

Reviewers' comments:

Reviewer's Responses to Questions

**Comments to the Author**

1. If the authors have adequately addressed your comments raised in a previous round of review and you feel that this manuscript is now acceptable for publication, you may indicate that here to bypass the “Comments to the Author” section, enter your conflict of interest statement in the “Confidential to Editor” section, and submit your "Accept" recommendation.

Reviewer #1: (No Response)

Reviewer #2: All comments have been addressed

2. Is the manuscript technically sound, and do the data support the conclusions?

The manuscript must describe a technically sound piece of scientific research with data that supports the conclusions. Experiments must have been conducted rigorously, with appropriate controls, replication, and sample sizes. The conclusions must be drawn appropriately based on the data presented.

Reviewer #1: Partly

Reviewer #2: Yes

3. Has the statistical analysis been performed appropriately and rigorously?

Reviewer #1: Yes

Reviewer #2: Yes

4. Have the authors made all data underlying the findings in their manuscript fully available?

The [PLOS Data policy](http://www.plosone.org/static/policies.action#sharing) requires authors to make all data underlying the findings described in their manuscript fully available without restriction, with rare exception (please refer to the Data Availability Statement in the manuscript PDF file). The data should be provided as part of the manuscript or its supporting information, or deposited to a public repository. For example, in addition to summary statistics, the data points behind means, medians and variance measures should be available. If there are restrictions on publicly sharing data—e.g. participant privacy or use of data from a third party—those must be specified.

Reviewer #1: Yes

Reviewer #2: Yes

5. Is the manuscript presented in an intelligible fashion and written in standard English?

PLOS ONE does not copyedit accepted manuscripts, so the language in submitted articles must be clear, correct, and unambiguous. Any typographical or grammatical errors should be corrected at revision, so please note any specific errors here.

Reviewer #1: Yes

Reviewer #2: Yes

6. Review Comments to the Author

Please use the space provided to explain your answers to the questions above. You may also include additional comments for the author, including concerns about dual publication, research ethics, or publication ethics. (Please upload your review as an attachment if it exceeds 20,000 characters)

Reviewer #1: Since the previous review round many of the questions have been addressed in this new version of the paper by Vettese et al., however, in some of the answers there are explanations only for the reviewer, but then actual changes or more explicit clarifications were not included in the paper.
Maybe something should be added to the text for these points too.

1) For example (NB line numbers, here as elsewhere, refer to the first version):

Reviewer:
Line 123 The experience of the observer/s should also be mentioned. Is the observer different from the person who analyzed the bones? This further character should be in case introduced

Authors:
The observers are different people with a basic knowledge of bone breakage. It was different from the people who analyzed the bones. However, all the breakage sessions were filmed, and the searcher who studied the bone remains watched them carefully to complete when it was necessary the data recorded during the experiments.

**Following the suggestion of the reviewer, we have added these sentences.**

Reviewer:
Line 187-188 The proposed “Efficiency Index calculated by dividing the mass of marrow extracted from each bone by the number of blows”, does not take into account the whole amount of marrow available in the bone that is of course dependent on the skeletal element (as also indicated by the authors later in the paper), maybe using in the index the ratio between the marrow extracted and the marrow available in that skeletal element instead of just the simple marrow extracted may help to overcome this problem.

Authors:
On this point, we would like to invite the reviewer to refer to a first report published online on PCI Archeology (see below).
“We are aware of this difference between the elements tested. However, we were able to observe among the different elements proposed to the same individual, there was a difference in size, in the quantity of spongiosa in the bone that could lead to a difference in the quantity of marrow. It was very complex without prior treatment to know the exact marrow content of each bone. For these reasons, we consciously choose to test the empirical hypothesis of whether the fact of intrinsically less accessible marrow having (as for the radius, where the marrow is contained in the proximal diaphysis for example) leads to a multiplication of the number of blows, of remains produced. It is, of course, possible to look at the amount of marrow extracted on average for each type of element, and to normalize it by an average capacity. However, we can observe that there is on average more marrow contained in the femur, but it is not the bone from which the most marrow has been extracted. And we think that is an important issue to explore further. While recalling that the experimenters were novices, and some before their first bone, they did not really know where the marrow was and therefore where to place the blows.”

**We have made this change. “Despite this difference between the elements tested, we observe differences between the bones even in the same individual series: there was a difference in size, in the quantity of spongiosa and in the quantity of marrow. The knowledge of the exact marrow content of each bone was complex to determine before the extraction and varied according the bones. For these reasons, we tested if the fact of intrinsically less accessible marrow having (as for the radius, where the marrow is contained in the proximal diaphysis for example) leads to a multiplication of the number of blows and of remains produced. Our results show that, even if there is on average more marrow contained in the femur, but it is not the bone from which the most marrow has been extracted. While recalling that the experimenters were novices, and some before their first bone, they did not really know where the marrow was and therefore where to place the blows.”**

Reviewer:
Lines 563-565 “..for two-thirds of the individual bone elements, the number of blows is higher than the number of percussion marks and more surprisingly, for one-third of the bone elements, the number of percussion marks is higher than the number of blows.” Although I understand your point, as it is written, from a simple mathematical point of view, it is obvious that if for 2/3 a>b, for the remaining 1/3 b>a, why should be the second one more surprising?

Authors:
Yes, it is. The surprise do not come from the count, but from the fact, the blow do not every time create a percussion mark, so the multiplicity of the percussion marks are not directly linked to the impact point. The counter blow due to the anvil could create percussion marks and one blow could create more than one percussion mark due to the irregularity of the hammerstone even if it is not modified. This need to be tested further.

**We have made this change. “The surprise does not come from the count, but from the fact, the blow do not every time create a percussion mark, so the multiplicity of the percussion marks are not directly linked to the impact point. The counter blow due to the anvil could create percussion marks. Besides, one blow could create more than one percussion mark due to the irregularity of the hammerstone, even if it is not modified. But this need to be further explored.”**

Reviewer:
Lines 570-71 In the explanation provided for the higher number of percussion marks vs. blows that “The use of an anvil or the hammerstone could explain the differences recorded between the number of marks and blows”, I may understand the role of the anvil in producing more percussion marks, but it is not clear to me what features of the hammerstone may increase the number of marks. Maybe explain more explicitly both.

Authors:
Regarding the anvil, the contact with it produces a counterblow, that could produce an additional percussion marks; the shape of the hammerstone which is round but could have irregularities, could also produce many pits.

**We have made this change. “Therefore, the use of an anvil could create supplementary marks during the blow that is the counterblow. The shape of the hammerstone, which is round but could have irregularities, could also produce many pits.”**

2) In the first line of the abstract the authors should refer more generally to the Palaeolithic and not only to the Middle Palaeolithic following the corrections already made in the main text.

**We have made this change.**

3) To the reviewer first comment on the sentence:
Lines 68-69 “The definition of a butchery tradition is a systematic and counterintuitive pattern shared by a same group”; as mentioned before why should a tradition be necessarily counterintuitive? Although a repeated “anomaly” in the breakage pattern may represent a tradition (i.e., a cultural trait overcoming common sense), if the aim is the optimization of marrow extraction the pattern recorded may follow common wisdom even within a tradition

The authors answer :“In absolute terms, we agree with the reviewer, but in the case of a transmitted systematic practice similar to an intuitive one, it would not be possible to distinguish it from a non-transmitted intuitive practice. So the question of the tradition as a practice in the Paleolithic can only be evidenced by differentiating it from the intuitive one. That is why we propose this definition of butchering traditions on Palaeolithic context”

However, this means that it is only possible to evidence “cultural anomalies”, and this is of course true in general, but one should not ignore, just because it is not possible to see them clearly, that “normal” cultural patterns following what the authors would consider as the result of intuition do exist and may be probably related to strategies for the optimization of resource acquisition. Furthermore, these “normal” cultural patterns may be the majority.

This topic needs to be discussed in the text.

**We have made this change. We agree with the reviewer that this could be discussed. Nevertheless, taking into account our current knowledge, it is and probably will be almost impossible to know and to discern what would be of the order of “normality” or “cultural anomalies”. That is why, we choose to not discuss this aspect in this present paper.**

4) The paper does not evidence enough the fact that this is only a preliminary study, part of a larger project. Furthermore, some statements (e.g., lack of influence of freezing/thawing on breakage) are based only on few cases, nevertheless the authors appear (too) sure of their findings.

The awareness that sill a lot needs to be done should appear more clearly in the text and the authors should explain why they decided to submit only this part of their project (i.e., why they consider this set of experiments as complete on their own).
At this preliminary stage of the project maybe more nuanced statements would be more appropriate.

**We agree with the reviewer and we have made some nuances, notably in the conclusion. And we have added lines 757-760: “This study is part of a larger project based on the long bone breakage that allowed testing further hypotheses. Despite the fact that our results look promising, they are still preliminary and it still a lot needs to be done.”**

**Regarding the sample chosen, the results previously presented in Stavrova et al. 2019 required us to expand our sample size of the series studied. We chose this number of three series for each of the four elements because they were complete. In addition, these series contained enough bones and traces with different characteristics that allow testing statistically the variables we had previously identified.**

5) Possibly if this project (and consequently the paper) included also “experienced bone breakers” the proposed results, if confirmed, would have had a more solid background.

**We agree with the reviewer that the experiment with experienced bone breakers need to be test. That is why we performed an experiment with expert experimenters, the results of these will be the subject of further publications which is part of our larger project. The paper is already long and present significant results that should be completed with complementary publication on the subject. We added this statement to clarify lines 757-758: “In addition, experiment performed with experts in long bone breakage will be completed this present study and its results.”**

6) The fact that the bones were defrosted when broken does not necessarily imply that there had been no effects, at a microscopic level, on bone structure produced by ice crystals, especially larger ones like those resulting from “homemade” (vs. industrial) freezing.

**We agree with the reviewer that why, lines 413-417, we added: “Besides, the bone frozen presented mixed fracture planes [52]. However, there does not seem to have any role on the distribution of impact marks and the percussion marks typology. The 20 elements (10 humerus and 10 radio-ulnas) that have been frozen do not show a divergent distribution pattern compared to those that have not been frozen”.**


In the end I still have problems with the concept of intuitiveness and for me "directly apprehended" is something different from intuition.
Probably, this “intuitiveness” concept adopted by the authors may be similar to the one employed for example for a new computer program or a new cell phone that may be immediately very easy to use, but still, even in this case, just consider that what may be intuitive and easy for a young person, more familiar with electronic devices, may not be so for a more aged individual; therefore, also “intuitiveness” may in many cases be culturally influenced.

**We understand the reviewer confusion; however we used the definition of the Merriam-Webster dictionary. Moreover, the protocol was made with this concept and to approach an intuitive practice without previous knowledge on the activity of long bone breakage to extract the marrow. We tried all along the paper, to explain and apply this concept.**

Reviewer #2: I am grateful to the authors for their effort in modifying the text to address the main weaknesses.
I think that in general the critical points have been solved, although there is still a minor critical point about the variables to control (especially, the state of the bone at the moment of breaking [e.g., frozen bones vs fresh bones?]) to solve. The authors in their answer state that the state of the bone does not affect the distribution of percussion marks ; however I think this statement (and a short explanation) should be clearly described in the text to make it clear, as it can be a contentious point when reading the study. If this clarification is added to the manuscript, I think it is ready for publication.
Thanks to the authors for this nice contribution.

**We agree with the reviewer and this comment is similar with the first reviewer that is why we added line 413-417: “Besides, the bone frozen presented mixed fracture planes [52]. However, there does not seem to have any role on the distribution of impact marks and the percussion marks typology. The 20 elements (10 humerus and 10 radio-ulnas) that have been frozen do not show a divergent distribution pattern compared to those that have not been frozen”.**

7. PLOS authors have the option to publish the peer review history of their article ([what does this mean?](https://journals.plos.org/plosone/s/editorial-and-peer-review-process#loc-peer-review-history)). If published, this will include your full peer review and any attached files.


If you choose “no”, your identity will remain anonymous but your review may still be made public.


**Do you want your identity to be public for this peer review?** For information about this choice, including consent withdrawal, please see our [Privacy Policy](https://www.plos.org/privacy-policy).

Reviewer #1: No

Reviewer #2: No

[NOTE: If reviewer comments were submitted as an attachment file, they will be attached to this email and accessible via the submission site. Please log into your account, locate the manuscript record, and check for the action link "View Attachments". If this link does not appear, there are no attachment files.]

While revising your submission, please upload your figure files to the Preflight Analysis and Conversion Engine (PACE) digital diagnostic tool, <https://pacev2.apexcovantage.com/>. PACE helps ensure that figures meet PLOS requirements. To use PACE, you must first register as a user. Registration is free. Then, login and navigate to the UPLOAD tab, where you will find detailed instructions on how to use the tool. If you encounter any issues or have any questions when using PACE, please email PLOS at [figures@plos.org](mailto:figures@plos.org). Please note that Supporting Information files do not need this step.

*In compliance with data protection regulations, you may request that we remove your personal registration details at any time.* [*(Remove my information/details)*](https://www.editorialmanager.com/pone/login.asp?a=r)*. Please contact the publication office if you have any questions.*

Haut du formulaire

Bas du formulaire
